# Efficacy and safety of acupuncture combined with Chinese herbal medicine in the treatment of angina pectoris of coronary heart disease (CHD): A protocol for systematic review and meta analysis

**DOI:** 10.1097/MD.0000000000028450

**Published:** 2021-12-30

**Authors:** Shuang Wang, Ai Dong Liu, Zhilei Wang, Yue Zhang

**Affiliations:** a Department of Traditional Chinese Medicine, Changchun University of Chinese Medicine, 1035 Boshuo Road, Changchun, Jilin, China; b Department of Cardiology, The Third Affiliated Clinical Hospital of Changchun University of Traditional Chinese Medicine, No. 1643 Jingyue Street, Jingyue High-Tech Development Zone, Changchun, Jilin, China.

**Keywords:** acupuncture, angina pectoris of coronary heart disease (CHD), Chinese herbal medicine, meta-analysis, systematic review

## Abstract

**Background:**

Coronary heart disease (CHD) angina pectoris is a clinical syndrome in which episodic chest pain or chest discomfort is the main manifestation of temporary ischemia and hypoxia of the myocardium due to coronary atherosclerosis and coronary artery functional changes (spasm). A large amount of clinical evidence confirms that acupuncture combined with Chinese herbal medicine in the treatment of CHD and angina pectoris can relieve the symptoms of angina pectoris and improve the performance of electrocardiograph ischemia; It still has obvious therapeutic effects in regulating the levels of cardiovascular regulatory peptides ET and cGRP. To better evaluate the effectiveness and safety of acupuncture combined with Chinese herbal medicine in the treatment of CHD and angina pectoris, we designed a systematic evaluation program to provide a reliable scientific basis for the future use of this method.

**Methods:**

Search Pubmed database, Embase, Cochrane library, Chinese Biomedical Literature CD-ROM Database (CBMdisk), China Journal Network Full-text Database (CNKI), Wanfang Database, Web of Science (SCI-E), the retrieval time is established from each database Until October 2021, search for relevant eligible randomized controlled trials with keywords or subject terms “acupuncture”, “Chinese herbal medicine”, and “CHD angina”. Outcome indicators were clinical symptoms of CHD and angina pectoris, changes in electrocardiogram, changes in blood lipids, and significant improvement in traditional Chinese medicine syndromes before and after treatment. Two researchers independently carried out data extraction and quality assessment, and use RevMan5.3 software to carry out final data analysis and assessment.

**Results:**

This study provides a reliable clinical scientific basis for acupuncture combined with Chinese herbal medicine for the treatment of CHD and angina pectoris.

**Conclusion:**

Acupuncture combined with Chinese herbal medicine can effectively relieve the clinical symptoms of CHD and angina pectoris and improve the performance of electrocardiograph. At the same time, it can reduce the cardiovascular regulatory peptide ET and increase the level of cGRP in the patient's plasma, thus confirming its effectiveness and safety.

## 1 Introduction

### 1.1 Coronary heart disease, angina pectoris and acupuncture

Coronary heart disease (CHD) angina pectoris is a cardiovascular disease with a common clinical morbidity and fatality rate that is increasing annually,^[1]^ which seriously endangers the lives and health of the people. From a pathological point of view, it is due to coronary atherosclerosis, which leads to an imbalance between coronary blood supply and myocardial ischemia and hypoxia, which is mainly manifested as a clinical syndrome of paroxysmal chest pain or chest discomfort.^[2]^ CHD and angina pectoris are stable and unstable. Clinically, nitrate ester preparations or β-receptor blockers are commonly used in the treatment of CHD and angina pectoris. By expanding the coronary arteries, increasing myocardial blood supply and oxygen, reducing heart rate and blood pressure, and reducing myocardial oxygen consumption, thereby alleviating the onset of angina pectoris.^[3]^ The symptoms of CHD and angina pectoris correspond to the categories of “chest pain”, “true heart pain”, and “jue heart pain” in Chinese medicine, which are recorded in ancient Chinese medicine books. The theory of traditional Chinese medicine (TCM) believes that the pathogenesis of this disease is based on deficiency and the deficiency is mixed with the deficiency. According to the actual symptoms of the disease, patients are divided into different syndrome types.^[4]^ Syndrome differentiation and treatment are performed according to different clinical manifestations, so that different Chinese herbal medicine prescriptions and external treatment methods are selected. With reference to related published literature,[^5^,^6^,^7^,^8^,^9^] acupuncture is an external treatment of TCM, which has the characteristics of safety, effectiveness, convenient operation, clear curative effect, and easy acceptance by patients. Acupuncture is used to treat CHD and angina pectoris. Under the guidance of TCM, it is regularly compatible to stimulate specific acupoints on the body's muscle surface, acting on the viscera and meridians to achieve a balance of qi, blood, yin, and yang in the body; acupuncture and moxibustion combined with Chinese herbal medicine can harmonize qi and blood, dredge the meridians, and relieve pain, so as to relieve the clinical symptoms of CHD and angina pectoris, improve the physical and chemical indicators, and achieve good clinical effects. But at present there is no clinical system evaluation and analysis of acupuncture combined Chinese medicine treating coronary heart disease angina pectoris the safety and effectiveness, this study suggests that the already published randomized clinical trials of acupuncture combined Chinese herbal medicine, a systematic review and meta-analysis to collect relevant evidence of safety and effectiveness of treatment, to provide reference for the clinical treatment method.

### 1.2 Protocol registration

The protocol for this systematic review has been registered on the INPLASY. Website (https://inplasy.com/inplasy-2021-11-0100/) and registration number: INPLASY202110100).

## 2 Materials and methods

### 2.1 Literature inclusion criteria

Clinically, it is clearly diagnosed as CHD and angina pectoris, and a randomized controlled trial using acupuncture and Chinese herbal medicine for treatment. There are no restrictions on the form of publication or language in the included literature.

### 2.2 Literature exclusion criteria

In the treatment of CHD and angina pectoris, the intervention measures did not use acupuncture combined with Chinese herbal medicine clinical randomized controlled trials, related animal experiments, review articles, etc.

### 2.3 Research objects

For patients who meet the clinical diagnostic criteria for CHD and angina pectoris, there are no restrictions on age, sex, course of disease, or geographic differences.

### 2.4 Intervention measures

The experimental group received acupuncture (including acupuncture, warm acupuncture, and other treatment methods) combined with Chinese herbal treatment, while the control group received conventional treatment for CHD and angina pectoris.

### 2.5 Outcome indicators

CHD angina pectoris clinical symptoms, angina pectoris duration, attack frequency, electrocardiogram performance, triglycerides, cholesterol indicators, and TCM syndrome scores.

### 2.6 Search strategies

#### 2.6.1 Literature sources

Electronic computer search is mainly for Pubmed database, Embase, Cochrane library, China Biomedical Literature CD-ROM Database (CBMdisk), China Journal Network Full-text Database (CNKI), Wanfang Database, Web of Science (SCI-E), and the search time is from each During the establishment of the database until October 2021, search related published literature with keywords such as “acupuncture”, “acupuncture”, “warm acupuncture”, and “CHD angina”, and screen the included literature in detail, such as If necessary, contact the author of the relevant literature.

#### 2.6.2 Retrieval method

**Figure 1:**
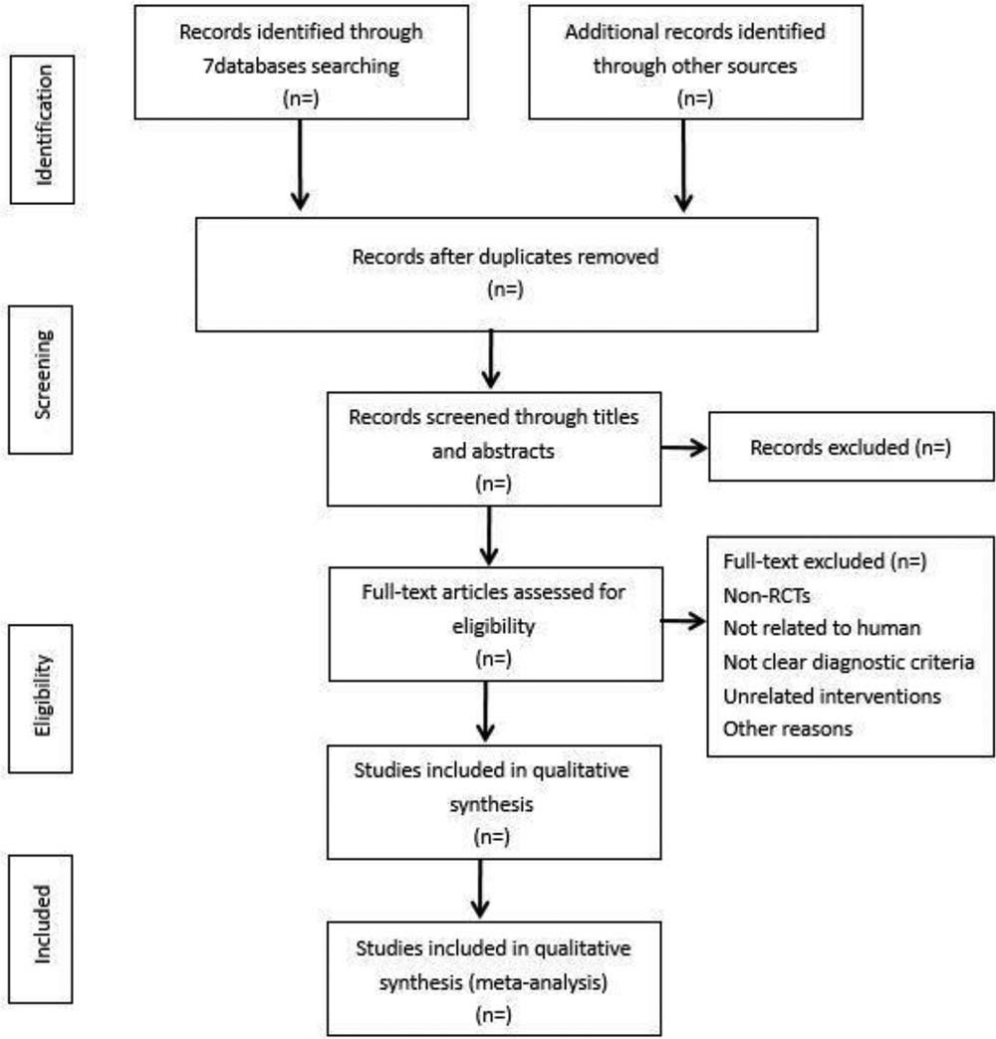
Flow diagram of study selection process.

According to the search strategy, 2 experienced researchers independently conducted related literature searches. By carefully reading the titles and abstracts of the literature, and according to the inclusion and exclusion criteria, the related literature was screened to determine whether it would be included in subsequent studies. If there is a dispute or a divergent issue, it will be resolved through internal consultation or discussion with a third party (Fig. 1, the screening process).

### 2.7 Data extraction

The following information was extracted step-by-step from the selected literature: ① basic information, that is, the title of the literature, author, and contact number; ② the specific situation of the research subjects included in the experiment, that is, gender, age, specific number of experimental groups, and time of participation; ③ intervention measures and outcome indicators, that is, acupuncture methods, acupoint compatibility, treatment time, and the name and dosage of the combined Chinese herbal medicine prescriptions; clinical symptoms of angina pectoris, duration of angina pectoris, attack frequency, electrocardiograph manifestations, triglycerides, cholesterol index, and TCM syndrome score.

### 2.8 Quality assessment/bias risk analysis

Use the evaluation tools provided in Cochrane Handbook 5.1.0 to conduct bias risk assessment, which includes the following aspects: namely, random sequence generation, allocation concealment, whether to use blind method, data integrity, selective reporting, other deviations, etc. The entire process was independently carried out by 2 researchers. If there is a disagreement in opinion during the evaluation process, we will adopt internal consultations or seek consultation with a third party to resolve.

### 2.9 Statistics analysis

RevMan5.3 software (Oxford, England) was used for the meta-analysis. The enumeration data adopted the risk ratio or odds ratio, and the measurement data adopts the mean difference or standardized mean difference. 95% CI is the efficacy analysis statistics; heterogeneity analysis: when *P* > .1, when *I* ^2^ > 50%, it indicates that there is heterogeneity, and determines the source of heterogeneity. Further subgroup analysis or sensitivity analysis was performed according to the clinical heterogeneity. If heterogeneity still exists, the random effects model is used.^[10]^

### 2.10 Subgroup analysis

As potential heterogeneity is inevitable, in order to reduce the impact of the final result, we performed subgroup analysis of all information collected in this study based on age, disease course, treatment type, treatment duration of all included subjects, and treatment of the control group.

### 2.11 Sensitivity analysis

Considering the impact of sensitivity analysis on methodological quality, we test the robustness of the results by excluding low-quality and high-bias risks.

### 2.12 Ethics and dissemination

Since the data in our study come from published literature and have nothing to do with the personal data of patients, ethical permission is not required; the final research results will be published in peer-reviewed journals and conference reports.

## 3 Discussion

With the deepening of theoretical research and understanding of TCM, the basic pathogenesis of CHD and angina pectoris is based on deficiency and excess, and deficiency and excess are mixed, and TCM therapy has made great progress in the treatment of patients with angina pectoris. Acupuncture, as an external treatment in Chinese medicine, has the characteristics of safety, greenness, and affirmative efficacy. It can effectively relieve the symptoms of angina during the treatment. However, we also found that in the actual clinical treatment process, although acupuncture points are selected under the guidance of TCM theory, there is a lack of corresponding judgment standards and operating specifications for acupuncture point selection, and there is a lack of further research on the effective stimulus amount and related mechanisms during actual operation.[^11^,^12^] Based on this, we should strengthen the randomized controlled study on the efficacy and safety of acupuncture combined with Chinese herbal medicine in the treatment of CHD and angina pectoris in future studies. Provide a strong scientific basis for the application of this method to clinical treatment. To provide a strong scientific basis for the application of this method to clinical treatment.

## Author contributions

**Conceptualization:** Shuang Wang, Ai Dong Liu.

**Data curation:** Zhi Lei Wang, Yue Zhang.

**Formal analysis:** Shuang Wang, Zhi Lei Wang.

**Funding acquisition:** Ai Dong Liu.

**Methodology:** Shuang Wang, Yue Zhang.

**Resources:** Ai dong Liu.

**Software:** Shuang Wang, Zhi Lei Wang.

**Writing – original draft:** Shuang Wang.

**Writing – review & editing:** Ai Dong Liu.
